# A Disposable Photovoltaic Patch Controlling Cellular Microenvironment for Wound Healing

**DOI:** 10.3390/ijms19103025

**Published:** 2018-10-04

**Authors:** Hyeon-Ki Jang, Jin Young Oh, Gun-Jae Jeong, Tae-Jin Lee, Gwang-Bum Im, Ju-Ro Lee, Jeong-Kee Yoon, Dong-Ik Kim, Byung-Soo Kim, Suk Ho Bhang, Tae Il Lee

**Affiliations:** 1 Research Institute for Convergence of Basic Sciences, Hanyang University, Seoul 04763, Korea; alwayshk@hanyang.ac.kr; 2 Department of Chemical Engineering, Kyung Hee University, Yongin 17104, Korea; jyoh@khu.ac.kr; 3 Division of Vascular Surgery, Samsung Medical Center, Sungkyunkwan University School of Medicine, Seoul 06351, Korea; jgj814@skku.edu (G.-J.J.); dikim@skku.edu (D.-I.K.); 4 School of Chemical Engineering, Sungkyunkwan University, Suwon 16419, Korea; leetj@skku.edu (T.-J.L.); lki1005@skku.edu (G.-B.I.); 5 School of Chemical and Biological Engineering, Seoul National University, Seoul 08826, Korea; ljr0518@snu.ac.kr (J.-R.L.); yoonjk87@snu.ac.kr (J.-K.Y.); 6 Interdisciplinary Program for Bioengineering, Seoul National University, Seoul 08826, Korea; alwayshk@snu.ac.kr; 7 Bio-MAX Institute, Institute of Chemical Processes, Seoul National University, Seoul 08826, Korea; 8 Department of BioNano Technology, Gachon University, Seongnam 13120, Korea

**Keywords:** cutaneous wound, cellular microenvironment control, electrical stimulation, organic photovoltaic

## Abstract

Electrical stimulation (ES) is known to affect the wound healing process by modulating skin cell behaviors. However, the conventional clinical devices that can generate ES for promoting wound healing require patient hospitalization due to large-scale of the extracorporeal devices. Herein, we introduce a disposable photovoltaic patch that can be applied to skin wound sites to control cellular microenvironment for promoting wound healing by generating ES. In vitro experiment results show that exogenous ES could enhance cell migration, proliferation, expression of extracellular matrix proteins, and myoblast differentiation of fibroblasts which are critical for wound healing. Our disposable photovoltaic patches were attached to the back of skin wound induced mice. Our patch successfully provided ES, generated by photovoltaic energy harvested from the organic solar cell under visible light illumination. In vivo experiment results show that the patch promoted cutaneous wound healing via enhanced host-inductive cell proliferation, cytokine secretion, and protein synthesis which is critical for wound healing process. Unlike the current treatments for wound healing that engage passive healing processes and often are unsuccessful, our wearable photovoltaic patch can stimulate regenerative activities of endogenous cells and actively contribute to the wound healing processes.

## 1. Introduction

Wound healing is a complex process associated with various types of cells, cytokines, and growth factors. Appropriate regulation of immune reactions and therapeutic manipulation of skin cells are crucial for the treatment of recalcitrant wounds, acceleration of wound healing, and minimization of scar tissues [1,2,3]. Among a series of endogenous healing mechanisms involved in wound repair, an endogenous electric field (EF), which ranges from 40 to 200 mV/mm, generated in the wound region, is known as a regulator of wound healing [4]. The EF stimulates the behaviors of not only the skin cells but also the immune cells that participate in the wound healing process by directing and enhancing migration of neutrophils [5,6], keratinocytes [5,7,8], and fibroblasts [5,9,10]. In addition, fibroblast proliferation, secretion of extracellular matrix (ECM) proteins [11], and fibroblast growth factors (FGF) 1 and 2 are enhanced by electrical stimulation [12].

Researchers have applied exogenous electrical stimulation to elucidate the therapeutic effects on wound healing. Even though previous animal studies and clinical trials have shown that the exogenous electrical stimulation is effective for wound healing [13,14], these studies utilized large devices to deliver electrical stimulation to an injury site, and the therapy required hospitalization of the patients. Therefore, the development of a wearable device that can deliver electric stimulation to the wound site can minimize the inconvenience of patient hospitalization and allow for more patient-friendly clinical application of the electrical stimulation therapy to wound healing is needed. Additionally, unlike current wound dressings that engage passive healing processes and focus on minimizing tissue infections and rehydrating the wound site [15], a wearable electric stimulation device can stimulate the regenerative activity of endogenous cells associated with the wound repair.

Here, we introduce a wearable, self-powered, and disposable organic photovoltaic patch (OPP) for electrically stimulated cutaneous wound healing therapy. Some studies show that direct current ranging between 200 and 800 µA has a positive effect on the outcome of treatments of chronic wounds in humans [16,17]. An OPP can generate direct current in hundreds of microamperes or electric field (40–200 mV/mm) that has an appropriate scale for cutaneous wound healing. We chose a conventional organic solar cell based on poly(3-hexylthiophene) (P3HT) and the phenyl-C_61_-butyric acid methyl ester (PCBM) bulk heterojunction as a wearable and disposable electric stimulation generator because an organic solar cell is mechanically flexible, biocompatible with skin, and mass-producible with low cost [18,19]. Our patch has two major parts: an organic solar cell and a patch with electrodes adjusted to the wound geometry. The solar cell and the electrodes are electrically connected, integrated, and packaged to be a single, wearable OPP. The outer circular electrode is designed to cover the outer wound area. The inner electrode is located in the center of the wound. Thus, electric stimulation generated by the organic solar cell is aligned along the direction of wound closing. The OPP can provide long-term electrical stimulation under visible light illumination until wound healing is completed.

In the present study, we tested whether OPP stimulates the regenerative activities of endogenous cells, showing wound healing efficacy superior to a conventional wound dressing when applied to cutaneous wounds on the back of mice. The OPP attached to a mouse’s back can receive light energy from a light emitting diode (LED) light in the cage and convert it into electrical stimulation that can be delivered to the wound through the gold (Au) electrodes. We first determined whether the OPP successfully converted light energy into direct current suitable for wound healing. Next, we tested whether the electrical stimulation promoted the regenerative activities of endogenous cells such as fibroblasts at the wound site by examining inflammation, re-epithelialization, proliferation, granulation, angiogenesis, and tissue remodeling. Finally, we assessed the wound healing promoted by the electrical stimulation with OPP as compared to treating conventional wound dressing.

## 2. Results

### 2.1. In Vitro Effects of Electrical Stimulation on Cell Migration, Cell Death, Proliferation, Cell Matrix Component Regulation, and Differentiation

**Figure 1:**
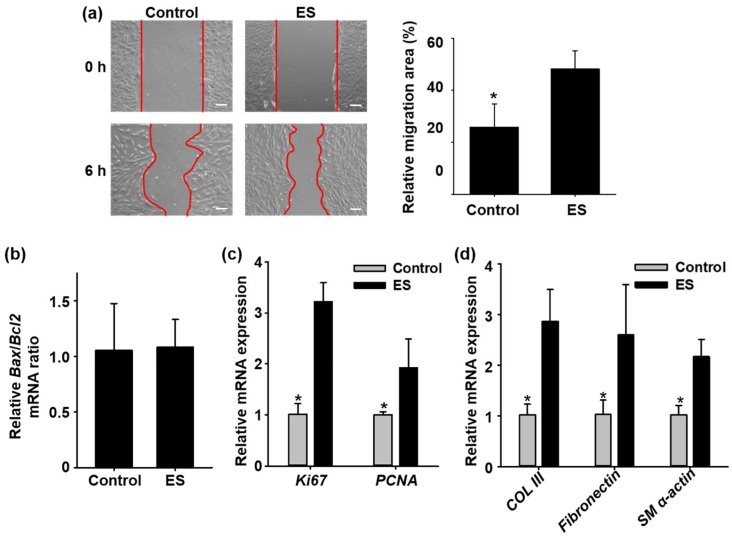
In vitro results showing effect of electrical stimulation on skin regeneration. (**a**) Enhanced fibroblasts migration after electrical stimulation (ES, 6 h) compared to no treatment (control). (**b**) Viability of fibroblasts after exposure to electrical stimulation for 6 h evaluated by pro-apoptotic gene (*Bax*) and anti-apoptotic gene (*Bcl2*) expression using quantitative real-time polymerase chain reaction (qRT-PCR). (**c**) Proliferation of fibroblasts following exposure to electrical stimulation for 6 h evaluated by *Ki67* and proliferating cell nuclear antigen (PCNA) expression using qRT-PCR. (**d**) Extracellular matrix (type III collagen (COL III) and *fibronectin*) and myofibroblastic differentiation (smooth muscle alpha actin (SM α-actin)) related messenger ribonucleic acid (mRNA) expression from fibroblasts following exposure to electrical stimulation for 6 h using qRT-PCR. * *p* < 0.05 compared to the ES group.

The effects of electrical stimulation on fibroblasts were investigated. Fibroblasts were cultured in the electric chamber and exposed to electrical stimulation (70 mV/mm) for 6 h. Electrical stimulation enhanced fibroblasts migration compared to the fibroblasts cultured without electrical stimulation (Figure 1a). However, electrical stimulation did not affect the migration of keratinocytes (data not shown). After electrical stimulation, the fibroblasts were analyzed with pro-apoptotic (*Bax*), anti-apoptotic (*Bcl2*), and proliferation (*Ki67* and proliferating cell nuclear antigen (*PCNA*)) related gene expression using quantitative real-time polymerase chain reaction (qRT-PCR). Electrical stimulation had no effect on the pro-apoptotic gene expression, as shown in Figure 1b. Proliferation related gene (*Ki67* and *PCNA*) expression levels in fibroblast were significantly increased by electrical stimulation (Figure 1c). Representative components of wound matrix, such as fibronectin and type III collagen (*COL III*), were upregulated in fibroblasts when the electrical stimulation was applied (Figure 1d). Relative messenger ribonucleic acid (mRNA) levels of smooth muscle alpha actin (*SM α-actin*), an early differentiation marker of smooth muscle cells, was also upregulated in the fibroblasts when electrical stimulation was applied (Figure 1d).

### 2.2. Fabrication and Characterization of the OPP Applied to Wound Healing

**Figure 2:**
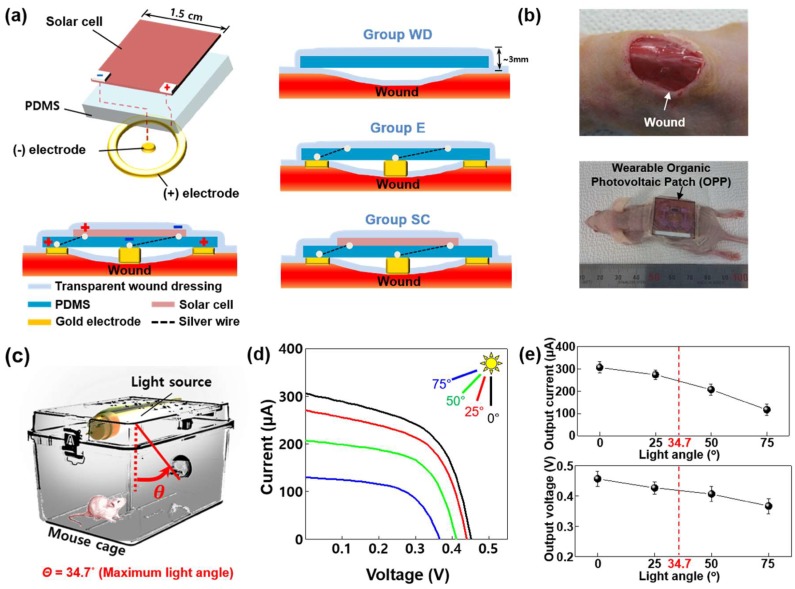
Characterization and the method of treating disposable organic photovoltaic patch (OPP) to mouse wound model. (**a**) Schematic illustration of manufacturing OPP and its application to mouse wound models. (**b**) Photographs of a mouse wound model with (upper panel) or without (lower panel) OPP treatment. (**c**) Illustration of the experimental environment for the wounded mouse that wears the OPP in the cage with light emitting diode (LED) light. (**d**) Photovoltaic capacity of the patch according to a change of the light angle. (**e**) A graph of the output current, output voltage, applied current, and applied voltage of the patch according to a change in the light angle.

Figure 2a shows the schematic illustration of the OPP applied to wound healing. The patterned three organic solar cells were fabricated on one substrate as a type of inverted structure that has better stability in air. The devices were electrically connected in parallel. Patches without solar cell/electrode (wound dressing, Group WD) or solar cell (electrode only, Group E) were also fabricated and used as controls. The molded circle electrodes on polydimethylsiloxane (PDMS) substrate were combined with the organic solar cell part (wearable OPP, Group SC). The patches were applied to the skin wounds of mice to test the healing effect, as shown in Figure 2b. Figure 2c shows the experimental design of light source control using LED light in mouse cage. The maximum angle between the light source and the photovoltaic cell was 34.7°. Figure 2d depicts current versus voltage (I–V) curves of the OPP under various light angles considering the mouse movements. Although open circuit voltage (V_oc_) and short circuit current (I_sc_) decreased with the increasing light angle because of reduced light intensity, the actual output voltage and current under the light followed the normal tendency and stayed above 0.4 V and 250 µA (34.7° was maximum angle between the light source and the photovoltaic cell). In addition, the applied voltage and current (that the patches actually deliver when attached to the wound) were calculated from the wound resistance (≈100 kΩ). The applied voltage was similar to that of the V_oc_. The applied current was ~4 µA (Figure 2e).

### 2.3. Improved Wound Healing Induced by the OPP

**Figure 3:**
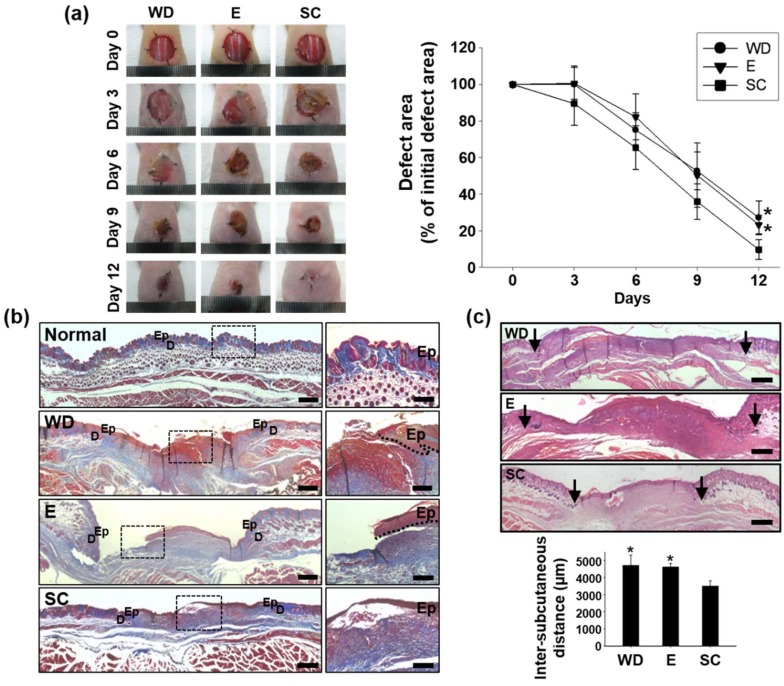

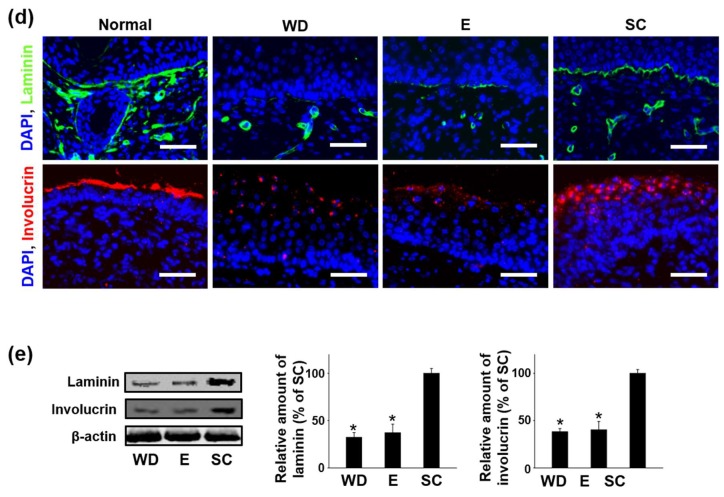
Acceleration of wound healing by the OPP treatment (WD: wound site treated only with the PDMS substrate, E: wound site treated with the electrode part that has no solar cells to generate electrical stimulation (electrode only), SC: wound site treated with OPP that generates electrical stimulation). (**a**) Representative photographs and quantification of the skin wound closure 0, 3, 6, 9, and 12 days after treatment (*n* = 8, * *p* < 0.01 versus SC). (**b**) Representative images of the Masson’s trichrome stained tissues sampled from the wound healing regions (Day 12, scale bars indicate 500 μm (left panel), 200 μm (right panel)). (**c**) Representative images of the hematoxylin and eosin stained tissues sampled from the wound healing regions (Day 12, scale bars indicate 500 μm, arrows in the figures indicate the margin of inter-subcutaneous distance) and the quantification of inter-subcutaneous distance (* *p* < 0.05 versus group SC). (**d**) Representative images of immunohistochemical staining with laminin (green, basal layer) and involucrin (red, epidermis) in the wound healing region 12 days after treatments. Scale bars indicate 50 μm. (**e**) Protein expression and quantification of laminin and involucrin in the wound beds 12 days after the treatments (Western blot analysis. * *p* < 0.05 versus group SC).

To evaluate the in vivo therapeutic effect of the OPP in mouse wound closing model, three groups including wound dressing (Group WD, wound site treated only with the PDMS substrate), electrode only (Group E, wound site treated with the electrode part that has no solar cells to generate electrical stimulation), and OPP (Group SC, wound site treated with OPP that generates electrical stimulation) were compared. The wounds in all groups were dressed with a transparent Tegaderm film to minimize tissue infections and to rehydrate the wound [15]. Digital photographs of the wound region were taken 0, 3, 6, 9, and 12 days after each treatment, and the relative wound area at each time point was expressed as the percentage relative to the initial wound size (Figure 3a). Group WD was served as negative control group. On day 6 and 9, all three groups showed a significant size reduction of wounds compared that of day 0. No statistical difference was observed in size reduction of wounds among groups until day 9 (Figure 3a). However, on day 12, group SC showed the smallest wound area compared to the other groups (Figure 3a). Group E showed no notable wound reduction compared to group WD. This result indicates that electrode itself has no therapeutic effect in wound repair and solar-cell-powered ionic current enhancement is critical for skin tissue regeneration. Masson’s trichrome staining at day 12 showed that there was more collagen deposition in group SC than in group WD and E (Figure 3b). OPP treatment enhanced re-epithelialization of the epidermis layer in the wound region, while group WD and E showed a rupture of the epidermis layer (Figure 3b).

Twelve days after the treatment, the inter-subcutaneous distance of group SC was significantly decreased compared to that of other groups (Figure 3c). Such a decrement in inter-subcutaneous distance was then accompanied with the enhanced regeneration of the basal layer (expression of laminin, Figure 3d,e) and epidermis layer (expression of involucrin, Figure 3d,e). Immunohistochemical staining showed significantly enhanced laminin, a biomarker for the skin basal layer, and involucrin, a representative biomarker of the skin epidermis layer, expression in group SC compared to other groups (Figure 3d). Similarly, western blot analysis of these biomarkers showed that the OPP treatment significantly promoted laminin and involucrin expression (Figure 3e).

### 2.4. Enhanced Regenerative Activities in the Wound Healing Process Induced by the OPP

**Figure 4:**
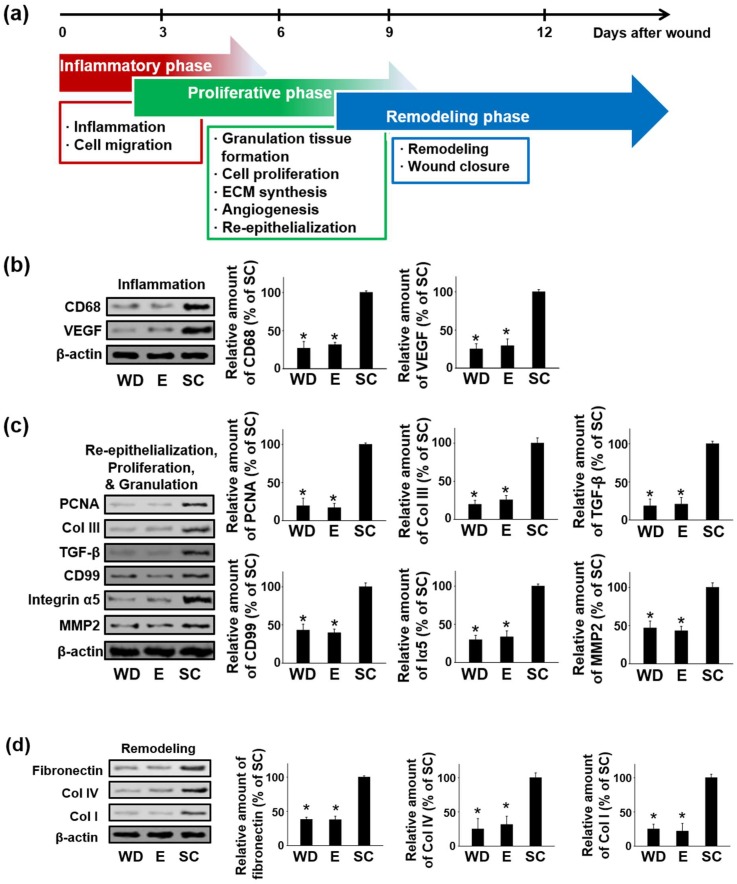

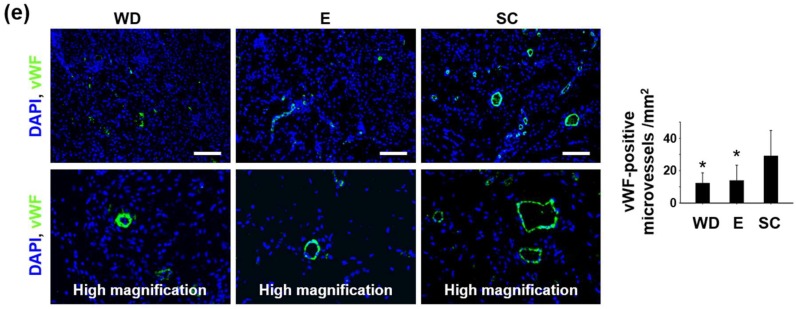
Enhanced regenerative activities in the wound healing process stimulated by the OPP treatment. (**a**) Schematic illustration of wound healing process. (**b**) Expression and quantification of the proteins related to the inflammatory phase (day 3) of the wound healing process. (**c**) Expression and quantification of the proteins contributing to the proliferative phase (day 9) of the wound healing process. (**d**) Expression and quantification of the proteins involved in the remodeling phase (day 12) of the wound healing process. (**e**) Immunohistochemical staining and quantification of Von Willebrand factor (vWF)-positive microvessels in the wound healing region 12 days after the treatments (scale bars indicate 100 μm, * *p* < 0.05 versus group SC).

We evaluated the wound healing process in phases enhanced by OPP treatment and determined whether electrical stimulation generated by the OPP treatment promoted each phase of wound repair process, namely the inflammatory phase, proliferative phase, and remodeling phase (Figure 4a). Inflammation, cell proliferation, and tissue remodeling phases of wound healing were assessed using Western blot analysis at 3, 9, and 12 days after the treatments, respectively (Figure 4b–d). During the inflammatory phase of wound repair, significant upregulation of CD68, inflammatory macrophage marker, expression was observed in group SC compared with other groups (Figure 4b). Moreover, vascular endothelial growth factor (VEGF) expression was also significantly upregulated by the OPP treatment in this phase (Figure 4b).

On day 9, tissue samples were retrieved and analyzed to evaluate the active proliferative phase at the wound site. As shown in Figure 4a, the proliferative phase consists of granulation tissue formation, cell proliferation, ECM synthesis, angiogenesis, and re-epithelialization. During this phase, significant upregulation of cell proliferation marker, PCNA, was observed (Figure 4c). Additionally, protein expression of COL III, transforming growth factor beta (TGF-β), an endothelial cell marker (CD99), integrin alpha-V subunit (Integrin α5), and matrix metalloproteinase 2 (MMP-2) increased significantly in group SC compared to other groups (Figure 4c). During the tissue remodeling phase, we observed notable upregulation of fibronectin, type IV collagen (COL IV), and type I collagen (COL I) in group SC compared to other groups (Figure 4d).

To further visualize therapeutic angiogenesis at the wound site under the influence of the OPP treatment, immunohistochemical staining for Von Willebrand factor, a microvessel biomarker, was performed. Twelve days after the treatments, OPP treated group showed enhanced microvessel formation in the wound region as compared to other groups (Figure 4e).

## 3. Discussion

In the present study, we introduced a disposable OPP for acceleration of wound healing by delivering electrical stimulation. First, we investigated the effect of electrical stimulation on cells in vitro (Figure 1). The strength of the electric field was 70 mV/mm, which was enough to enhance regenerative activity of fibroblasts [11,12]. Electrical stimulation by OPP was not cytotoxic (Figure 1b) and enhanced migration, proliferation, and ECM protein synthesis of fibroblasts (Figure 1a,c–d). Indeed, we confirmed electrical stimulation induced myofibroblastic differentiation of fibroblasts (Figure 1d). Migration of fibroblasts is an essential step in wound healing because the cells are involved in ECM production and contraction of wound size [12]. In addition, during the wound healing process, myofibroblasts reduce wound size through contraction of the actin cytoskeleton and organized ECM [20]. Thus, the increased differentiation into myofibroblasts may have a beneficial effect on wound closure. Collectively, these data indicate the therapeutic potential of an exogenous electrical stimulation for wound healing in vivo. In accordance with the in vitro experiments, the applied voltage of the OPP was about 0.45 V (Figure 2e), which generates 82 mV/mm electric field. The applied current was ~4 µA: much smaller than the current (200–800 µA) that has been proven to effective for chronic wound healing in humans [21]. Recently, however, Sara Ud-Din reported that electrical stimulation with a degenerative wave form delivering 4 µA of current was effective for cutaneous wound healing in humans [22]. This finding indicates the validity of our patch for treatment of the cutaneous wound.

The OPP accelerates wound closure when attached to mouse skin wounds. We found that collagen deposition was increased in the OPP-treated mice compared to results from group WD and E (Figure 3b). Formation and deposition of collagen fibers are particularly important for wound repair because collagen is necessary for granulation tissue organization and tissue membrane regeneration during the wound healing process [23]. Additionally, more collagen observed in Group SC showed that electrical stimulation generated by the OPP may have affected collagen synthesis by the fibroblasts, as observed in previous study [24]. These results suggest that OPP-derived electrical stimulation treatment can expedite collagen synthesis in the wound lesion and better regenerate the basal and epidermis layer to reduce the wound defect area.

Further analysis of wound region at the different wound healing phases elucidated the effects of the electrical stimulation on wound healing process. In the inflammatory phase, VEGF expression was enhanced in group SC compared to that of group WD and E (Figure 4b). VEGF, which can be derived from neutrophils and macrophages, is known to be strongly upregulated during the inflammatory phase and contribute to wound healing by recruiting inflammatory macrophages to the wound region [25,26]. Hence, enhanced macrophage expression along with VEGF during the initial phase of the wound healing process seemed reasonable. Recruitment of inflammatory macrophages is important because newly recruited macrophages can secrete a variety of cytokines involved in angiogenesis [27], clearance of cell debris from the wound site, fibroblast proliferation, and collagen synthesis [28]. In the proliferative phase, enhanced cell proliferation was observed in the wound bed treated with the OPP in accordance with the result of previous study [29]. This result suggests that active cell proliferation and tissue formation were occurring during OPP treatment. In addition to proliferation, various types of proteins were upregulated in the wound bed treated with the OPP (Figure 4c). Among these proteins, TGF-β, CD99, and Iα5 have been previously shown to be critical for vascular development and therapeutic angiogenesis [23,30,31]. After the inflammatory phase, significant angiogenesis is required at the wound region to expedite granulation tissue formation and ECM synthesis [23,30]. New blood vessels at the wound site can provide more nutrients and blood to the newly growing tissues, improving new tissue formation [23,30]. MMP-2, which plays a crucial role in the breakdown and re-establishment of ECM in tissue remodeling, is known to be highly expressed during the granulation tissue formation [32]. Thus, significant upregulation of MMP-2 in group SC (Figure 4c) confirmed that more granulation tissue was formed in the group treated with the OPP compared with the other groups. Enhanced COL III expression (Figure 4c), which is important for basement membrane regeneration in the wound repair process [23], also denotes the therapeutic efficacy of solar-cell-powered electrical stimulation in cutaneous wound healing. Finally, in the remodeling phase, upregulation of ECM proteins such as fibronectin, COL I, and COL IV was observed in the wound bed treated with the OPP (Figure 4d). Fibronectin plays a crucial role in capillary formation, thus participating in granulation tissue reorganization and basement membrane formation during wound healing [33]. Moreover, enhanced expression of COL IV in group SC (Figure 4d) may improve keratinocyte proliferation and expedite membrane reconstitution for wound healing, as shown in another study [34]. Additionally, increased COL I protein expression (Figure 4D) indicates that OPP treatment accelerated wound maturation compared to the other groups [23]. In addition, blood vessel formation was increased by the OPP treatment, which might be a result of enhanced VEGF (Figure 4b) or fibronectin (Figure 4d) expression [25,27,33].

Overall, these data suggest that the OPP contributed to every stage of the wound healing process by regulating wound inflammation, accelerating cell proliferation and granulation tissue formation, and completing tissue remodeling. OPP treatment does not involve exogenous drug delivery and may serve as a more patient-friendlier therapeutic modality for wound healing.

## 4. Materials and Methods

### 4.1. Cell Culture

Human dermal fibroblasts and human neonatal keratinocytes were purchased from Lonza Inc. (Rockland, ME, USA). Human dermal fibroblasts were cultured in Dulbecco’s modified Eagle’s medium (DMEM, high glucose, Gibco-BRL, Gaithersburg, MD, USA) that was supplemented with 10% (*v*/*v*) of fetal bovine serum (FBS, Gibco-BRL), 100 U/mL penicillin (Gibco-BRL), and 100 μg/mL streptomycin (Gibco-BRL). Keratinocytes were cultured in the keratinocyte growth medium (KGM-Gold^TM^, Lonza) that was supplemented with 100 U/mL penicillin (Gibco-BRL) and 100 μg/mL streptomycin (Gibco-BRL).

### 4.2. Electrical Stimulation of Cells In Vitro

An electric chamber was manufactured according to a previous study [35]. Human dermal fibroblasts or keratinocytes were plated on a glass substrate in the electric chamber and were allowed to adhere to the substrate for one day. A linear power supply unit (GPC-1850D, Good Will Instrument, New Taipei City, Taiwan) was used to run a current through the chamber. For the analysis of cell migration, scratches were made on each monolayer of cells, and the culture medium was changed. Then, 0.01 A/cm^2^ of electric current was applied to the electric chamber for 6 h, corresponding to a 70-mV/mm electric field in the chamber [35]. The cells were photographed by optical microscope (IX71, Olympus, Tokyo, Japan) before and after the electrical stimulation. The cells were cultured 18 h further without electrical stimulation for gene expression analysis.

### 4.3. qRT-PCR

qRT-PCR was used to quantify the relative gene expression levels of *BAX*, *BCL2*, *Ki67*, *PCNA*, *COL III*, *fibronectin*, and *SM α-actin* (*n* = 4). Total RNA was extracted from samples using TRIzol. The RNA pellet was washed with 75% (*v*/*v*) ethanol in water and dried. After drying, the samples were dissolved in RNase-free water. For qRT-PCR, the iQ™ SYBR Green Supermix Kit (Bio-Rad, Hercules, CA, USA) and the MyiQ™ single color real-time PCR detection system (Bio-Rad) were used. β-actin served as the internal control.

### 4.4. Organic-Solar-Cell Fabrication

Organic solar cells were fabricated as described in our previous study [18]. Zinc oxide (ZnO) as an electron transport layer was deposited by the sol-gel method on a patterned ITO-glass substrate. A ZnO/ITO-glass substrate was spin-coated with a photoactive solution (P3HT:PCBM, 2 wt%, 1:1 *w*/*w*, in m-xylene). The photoactive layer (150 nm) was then spin-coated with a PEDOT:PSS layer (40 nm) as hole transport layer, and the substrate was thermally annealed at 150 °C for 10 min in a N_2_ atmosphere. A silver (Ag) electrode was thermally evaporated on the PEDOT:PSS layer as a top electrode in high vacuum (low 10^−6^ torr). Individual three photovoltaic devices with 16 mm^2^ were connected in parallel in a substrate. The organic solar cells were finally encapsulated by epoxy for ambient stability with a metal wire to connect with wound healing electrodes.

### 4.5. Preparation of Wound Healing Electrodes and Patch Integration

Two concentric-circle electrodes plated with Au were molded on the surface of a PDMS elastomer substrate with a metal wire to connect with the cathode and anode of the organic solar cell. The inner one is located in the center of PDMS substrate diameter and has a 4-mm diameter. The outer one has a 15-mm inner circle diameter and 3-mm thickness. The organic solar cell and wound healing electrode were connected by metal wire welding. The two parts were integrated by filling the gap of two parts with a PDMS elastomer.

### 4.6. Characterization

The photovoltaic characteristics of the patch were measured using a Keithley 2400 source measurement device under white LED light (DI-LED-6W, SFS Lights, Guangzhou, China) in air.

### 4.7. Treatment of Cutaneous Wound Bed

Six-week-old female athymic mice (20–25 g body weight, Orient, Seoul, Korea) were anesthetized with xylazine (20 mg/kg) and ketamine (100 mg/kg). A 12-mm round full-thickness excisional wound was made on the dorsal back of each mouse. Epidermis, dermis, subcutaneous tissue, and panniculus carnosus were removed, and the muscle tissue was exposed. Four knots (6-0 sutures; Ethicon, Somerville, NJ, USA) were tied at the boundary of the wound. The mice were randomly assigned to one of three treatments as follows: wound dressing (Group WD), electrode only (Group E), or wearable OPP (Group SC). The wound lesion of group WD was dressed with a transplant Tegarderm film (Tegarderm^TM^, 3M Health Care, St. Paul, MN, USA), and the PDMS substrate was attached to the wound site. The wound lesion of Group SC was dressed with the Tegarderm film (3M Health Care) and treated with the wearable OPP. The electrode directly contacted a wound lesion or normal skin of the border zone to deliver an electrical signal to the wound. The wound lesion of group E was also dressed with the Tegarderm film (3M Health Care) and treated with the electrode part that has no solar cells to generate electrical stimulation. The electrode directly contacted a wound lesion or normal skin of the border zone. After the treatments, the wounds in all groups were dressed again with the Tegaderm film (3M Health Care) to attach the device to the wound site, while minimizing tissue infection and rehydrating the wound [15]. To provide a stable light source for the solar cells, a bar-shaped LED lamp (SFS Lights) was installed in all cages. The lamps were set up to switch on/off in 12-h periods according to the “Guide for the Care and Use of Laboratory Animals” of Seoul National University. Wound healing status was followed up to 12 days after the treatment. Every three days, the Tegaderm film (3M Health Care) was replaced with a new one, and the electrode part was cleaned with ethanol-soaked gauze. The wound lesion was wet by sterile phosphate-buffered saline-soaked gauze to prevent drying while the Tegaderm film (3M Health Care) was replaced. All samples were collected in an identical manner to compare the wound healing among the groups. Entire tissues in the dorsal wound area were excised for analyses to compare the wound healing processes that occurred in the wound area among the groups. All animals received care according to the “Guide for the Care and Use of Laboratory Animals” of Seoul National University. This animal study was performed with permission from the Institutional Animal Care and Use Committee at Seoul National University (No. SNU-160304-6, 4 March 2016).

### 4.8. Histology

The tissues of the wound regions were retrieved 12 days after the treatments. The tissues were fixed in 10% (*v*/*v*) buffered formaldehyde, dehydrated using a graded series of ethanol solutions, embedded in paraffin, and sliced into 4-μm thick sections. Microscopic tissue regeneration was evaluated using Masson’s trichrome- or hematoxylin and eosin- stained tissue slices and a light microscope (KS400, Zeiss). The inter-subcutaneous distance was measured (four different samples per group) on digital images using imaging software ImageJ (NIH, Bethesda, MD, USA).

### 4.9. Morphometric Analysis

The macroscopic wound area was quantified by processing photographs obtained at various time points (Day 0, 3, 6, 9, and 12) by tracing the wound margin and calculating the pixel area. The location of the advancing margin of wound closure was defined as the grossly visible margin of epithelial migration toward the center of the wound and over the granulation tissue bed. The wound area was calculated (eight different samples per group) as the percentage of the initial wound area ([wound area at time] ÷ [initial wound area] × 100%). Morphometric analysis was performed based on the digital images using the imaging software ImageJ (NIH).

### 4.10. Immunohistochemistry

The tissues of the wound site were retrieved 12 days after the treatment and embedded in an optimal cutting temperature compound (TISSUE-TEK^®^ 4583, Sakura Finetek USA Inc., Torrance, CA, USA), followed by freezing and slicing into 10-μm thick sections at −22 °C. Immunohistochemical analysis of laminin (Abcam, Cambridge, UK) and involucrin (Abcam) was performed to examine the regeneration of the basal layer and epidermis, respectively. The staining signals for laminin and involucrin were visualized using fluorescein isothiocyanate-conjugated or rhodamine-conjugated secondary antibodies (Jackson Immuno Research Laboratories, West Grove, PA, USA), respectively. To assess the microvessel density, tissue slices were stained with antibodies against vWF (Abcam). vWF-positive signals were visualized using fluorescein isothiocyanate-conjugated secondary antibodies (Jackson Immuno Research Laboratories). The tissue slices were mounted in 4′,6-diamidino-2-phenylindole (DAPI, Vector Laboratories). A fluorescent microscope (Olympus) was used to count the microvessels. Four different images per slide from 20 random slides were randomly analyzed for each group (four different samples per group) for immunohistochemical quantification.

### 4.11. Western Blot Analysis

Tissue samples were obtained from the mouse wound area 12 days after the treatments, and homogenized using a Dounce homogenizer (50 strokes, 4 °C) in ice-cold lysis buffer (15 mM Tris-HCl, pH 8.0, 0.25 M sucrose, 15 mM NaCl, 1.5 mM MgCl_2_, 2.5 mM ethylenediaminetetraacetic acid, 1 mM ethylene glycol tetraacetic acid, 1 mM dithiothreitol, 2 mM NaPPi, 1 mg/mL pepstatin A, 2.5 mg/mL aprotinin, 5 mg/mL leupeptin, 0.5 mM phenylmethyl sulfonyl fluoride, 0.125 mM Na_3_VO_4_, 25 mM NaF, and 10 mM lactacystin). The protein concentration was determined using a bicinchoninic acid protein assay (Pierce Biotechnology, Rockford, IL, USA). Western blot analysis was performed using sodium dodecyl sulfate polyacrylamide gel electrophoresis in a 10% gel. After the proteins were transferred onto an Immobilon-P membrane (Millipore Corp., Billerica, MA, USA), they were probed with antibodies against laminin, involucrin, caspase 3, CD68, mouse vascular endothelial growth factor (VEGF), PCNA, COL III, transforming growth factor beta (TGF-β), CD99, Iα5, matrix metalloproteinase 2 (MMP-2), fibronectin, COL IV, COL I, and β-actin (all antibodies were purchased from Abcam) and incubated with a horseradish peroxidase-conjugated secondary antibody (Santa Cruz Biotechnology, Santa Cruz, CA, USA) for 1 h at room temperature. The blots were developed using an enhanced chemiluminescence detection system (Amersham Bioscience, Piscataway, NJ, USA). Luminescence was recorded on an X-ray film (Fuji super RX, Fujifilm Medical Systems, Tokyo, Japan), and the bands were imaged and quantified (four different samples per group) using an Imaging Densitometer (Bio-Rad).

### 4.12. Statistical Analysis

All quantitative data are expressed as mean ± standard deviation. A one-way analysis of variance (ANOVA) with the Bonferroni test was performed to determine significant differences. The assumptions of ANOVA were found to satisfy Levene’s test for homogeneity of variance and to pass tests for normality. A value of *p* < 0.05 was considered statistically significant.
